# Mechanisms underlying purinergic P2X3 receptor-mediated mechanical allodynia induced in diabetic rats

**DOI:** 10.1186/1744-8069-7-60

**Published:** 2011-08-18

**Authors:** Guang-Yin Xu, Guangwen Li, Ningang Liu, Li-Yen Mae Huang

**Affiliations:** 1 Institute of Neuroscience and Department of Neurobiology and Psychology, Key lab of Pain Research and Therapy, Soochow University, Suzhou 215123, the People's Republic of China; 2 Division of Gastroenterology, Department of Internal Medicine, University of Texas Medical Branch, Galveston, Texas 77555-0655, USA; 3 Department of Neuroscience and Cell Biology, University of Texas Medical Branch, Galveston, Texas 77555-1069, USA

## Abstract

**Background:**

Diabetic neuropathy is a common neuropathy associated with paresthaesia and pain. The mechanisms underlying the painful conditions are not well understood. The aim of this study is to investigate the participation of purinergic P2X3 receptors in painful diabetic neuropathy.

**Results:**

Diabetes was induced by an intraperitoneal injection of streptozotocin (STZ). We showed that mechanical allodynia was induced two weeks after a STZ injection and lasted for at least another seven weeks. The mechanical allodynia was significantly attenuated by peripheral administration of the P2X receptor antagonists, PPADS or TNP-ATP. DiI was subcutaneously injected into the rat hindpaw to label hindpaw-innervated dorsal root ganglion (DRG) neurons. ATP activated fast-inactivating P2X3 receptor-mediated currents in the labeled DRG neurons were studied. ATP responses in STZ-treated rats were ~2-fold larger than those in control rats. Furthermore, the expression of P2X3 receptor proteins in the plasma membrane of L4-6 DRGs of STZ rats was significantly enhanced while the total expression of P2X3 receptors remained unaltered.

**Conclusions:**

These results indicate that a large enhancement of P2X3 receptor activity and an increase in the membrane expression of P2X3 receptors contribute to the development of chronic pain in STZ-induced diabetic rats and suggest a possible target for the treatment of diabetic neuropathic pain.

## Background

Diabetics mellitus is a debilitating chronic disease that affects ~8% of the population in the US. About ~70% of diabetic patients are reported to have various forms of nerve damage (neuropathy). The most common type of diabetic neuropathy is nerve damages in the periphery, e.g., hands, toes and feet. Peripheral neuropathy patients often experience aberrant pain sensation, including spontaneous pain, hyperalgesia (severe pain with mild painful stimuli) and allodynia (pain with innocuous stimuli, e.g. light touch) [1-6]. Treatment options for these abnormal sensations have been limited, partly because of our poor understanding of the molecular mechanisms underlying the diabetes-induced neuropathic pain.

Sensitization of dorsal root ganglion neurons and their associated nerve fibers has been suggested to be a major cause of diabetes-induced abnormal pain [4,7]. Changes in the expression or function of T-type Ca^2+ ^channels [7] and transient receptor potential vanilloid 1 (TRPV1) receptors [6,8] are thought to contribute to changes in the activity of sensory neurons under diabetic conditions.

P2X receptors (P2XRs) are ligand-gated cationic channels abundantly expressed in the pain processing DRG neurons [9-11]. There is growing evidence that P2XR expression and function in DRGs are greatly sensitized after tissue inflammation [12,13] and nerve injury [14]. Thus, P2XRs play a pivotal role in the transmission of nociceptive information under injury conditions [9,15,16]. ATP and noradrenaline are co-stored and co-released from sympathetic nerves [17]. EctoATPase, which hydrolyzes extracellular di- and triphosphate nucleotides, modulates purinergic transmission [17]. It has been established that ATP and purinergic receptors are involved in peripheral signaling in diabetic rats. Stimulation of sympathetic nerves in those rats causes C-fiber polymodal receptor units to fire [18]. An increase in the extracellular level of adenosine by an adenosine kinase inhibitor attenuates diabetes-induced tactile allodynia [19]. Studying the involvement of P2XRs in painful diabetic neuropathy, Migita et al (2009) [20] found that P2X receptor antagonists inhibit the STZ-induced mechanical allodynia in mice and the levels of P2X2R and P2X3R mRNA in DRGs are increased. The molecular mechanism underlying the change in P2XR-mediated activity, however, has not been investigated. We therefore studied P2X3R current activity of hindpaw innervated DRG neurons in STZ-induced diabetic rats. Total and membrane expressions of the P2X3R protein were also investigated. We found that following the development of diabetes, P2X3R-mediated current activity is greatly enhanced and the membrane, but not the total, expression of P2X3Rs is significantly upregulated.

## Materials and methods

### Induction of diabetes

All experiments were approved by the Institutional Animal Care and Use Committee at the University of Texas Medical Branch and were in accordance with the guidelines of the International Association for the Study of Pain. Male Sprague-Dawley rats weighing 190-220 g were used in our experiments. Diabetes were induced by a single injection of streptozotocin (STZ; 70 mg/kg i.p., Sigma Chemicals, St. Louis, MO), which was freshly dissolved in 0.9% sterile saline as described [4,21-23]. Two weeks later, diabetes was confirmed by measurements of blood glucose concentrations in samples obtained from the tail vein. Only rats with blood glucose concentration > 350 mg/dl and mechanical paw withdrawal threshold (PWT) < 5 g were further used in the study.

### Cell labeling

Hindpaw receptive field specific DRG neurons were labeled by injection of 1,19-dioleyl-3,3,39,3-tetramethylindocarbocyanine methanesulfonate (DiI; Invitrogen, Carlsbad, California) into the skin of rat hindpaws. Animals were anesthetized with a cocktail of ketamine (80 mg/kg) and xylazine (5-10 mg/kg, intraperitoneally). DiI (25 mg in 0.5 ml methanol) was injected into the plantar skin of bilateral hindpaws (2 μl/site, 4-6 sites in each hindpaw). To prevent leakage, needle was left in place for 1 min for each injection. Two weeks later, bilateral L4-L6 DRGs in control and STZ rat groups were dissected out for patch clamp recordings.

### Whole-cell patch clamp recordings

As described previously [24], L4-L6 DRGs were dissected out and incubated in oxygenated dissection solution with enzymes (collagenase D, 1.5-1.8 mg/ml and trypsin, 1.2 mg/ml; Sigma) for 1.5 hr at 34.5°C. The dissection solution contained (mM): NaCl 130; KCl 5; KH_2_PO_4 _2; CaCl_2 _1.5; MgSO_4 _6; glucose 10; and HEPES 10; pH 7.2; osmolarity 305 mOsm. DRGs were then taken from the enzyme solution, washed, and transferred to the dissection solution containing DNase (0.5 mg/ml). A cell suspension was subsequently obtained by repeated trituration through a series of flame-polished glass pipettes. Cells were then plated onto acid-cleaned glass coverslips. One coverslip containing adherent DRG cells was put into a small recording chamber (0.5 ml volume) attached to the stage of an inverting microscope (Olympus IX71, Tokyo, Japan), which was fitted with both fluorescent and phase objectives. DiI-labeled neurons were identified by the bright red fluorescence in the cytoplasm. ATP-evoked currents were recorded in the external solution, containing (mM): NaCl 130, KCl 5, KH_2_PO_4 _2, CaCl_2 _2.5, MgCl_2 _1, HEPES 10, glucose 10 (pH = 7.2, adjusted with NaOH, osmolarity = 295-300 mOsm). Unless indicated, patch-clamp pipettes had a resistance of 3-5 MΩ when filled with the pipette solution containing (mM): potassium gluconate 140, NaCl 10, HEPES 10, glucose 10, BAPTA 5 and CaCl_2 _1 (pH = 7.25 adjusted with KOH; osmolarity = 295 mOsm). ATP-induced currents were filtered at 2-5 kHz and sampled at 50 or 100 μsec per point.

### Measurement of hindpaw withdrawal threshold

Experiments were performed on STZ-treated rats and age-matched control rats. The mechanical threshold was determined before and after STZ or saline injection. To quantify the mechanical sensitivity of the hindpaw, rats were placed in individual plastic boxes and allowed to acclimate for > 30 min. Rat hindpaw PWTs in response to the stimulation of Von Frey filaments were determined. A series of calibrated von Frey filaments (ranging from 0.4 to 15.0 g) were applied perpendicularly to the plantar surface of the hindpaw with a sufficient force to bend the filaments for 60s or until paw withdrew. In the presence of a response, the filament of next lower force was applied. In the absence of a response, the filament of next greater force was applied. To avoid injury during tests, the cutoff strength of the von Frey filament was 15 g. The tactile stimulus producing a 50% likelihood of withdrawal was determined using the "up-down" method [25]. Each trial was repeated 2-3 times at approximately 2 min intervals. The mean value was used as the force produced a withdrawal response. All behavioral studies were performed under blind conditions.

### Drug application

The P2XR antagonist, PPADS and TNP-ATP (Sigma, St. Louis, MO) were used in this study. PPADS (25 mmol) or TNP-ATP (50 mmol) in a volume of 50 μl was subcutaneously injected to the plantar surface of a rat hindpaw. For control, the same volume of phosphate balanced saline (PBS), instead of an antagonist, was injected. Following the injection, one PWT measurement was obtained every 10 min for a period of 90 min. For patch clamp recordings, ATP (20 μM) was pressure delivered (1-2 psi) to the recorded cell through a two-tube applicator. One tube contained the ATP or ATP+antagonist solution and the other tube contained a wash solution devoid of ATP. Each tube was connected to a solenoid valve, which was controlled by computer pulses to start and stop the solution flow. The exchange rate was fast and would not limit peak ATP responses [12].

### Western blotting analysis

The expressions of P2X3Rs in L4-L6 DRGs from control and STZ-treated rats were measured using Western blot analyses [12]. The rats were euthanized by an overdose of pentobarbital. Lumbar DRGs from both sides of spinal cord were quickly dissected out and put into oxygenated dissection solution. To determine the expression of P2X3Rs on cell surface, DRGs were incubated with Sulfo-NHS-LC-Biotin (1 mg/ml, Pierce) for 30 min on ice as described [24]. Since biotin is impermeable to the cell membrane, only proteins on the cell surface were biotinylated. The unbound biotin in the solution was removed by 5 × wash of DRGs. DRGs were then homogenized in buffer A (Pierce) and centrifuged at 13,500 × *g *(4°C) for 12 min. A 200 μg protein sample was incubated with streptavidin beads (20 μl, Sigma) for 3 h at 4°C. The beads were washed 3 × with RIPA buffer and precipitated by centrifugation and collected. Sample buffer (50 μl) was added to the collected beads and boiled for 3 min. Beads were pelleted again by centrifugation and the supernatant was collected. The supernatant was diluted to the same volume as the starting material (i.e., 200 μg total protein). Equal volume of total and membrane samples was applied to SDS-PAGE. Membranes were incubated with various primary and secondary antibodies. Antibodies used were anti-P2X3Rs (1:1000 Neuromics, Minneapolis) and anti-extracellular signal-regulated kinase 1 (anti-ERK1) (1:1500, Pierce). Immunoreactive proteins were detected by enhanced chemiluminescence (ECL kit, Amersham-Pharmacia Biotech). The densities of protein bands were analyzed using NIH image software.

### Data analysis

All data were expressed as mean ± SEM. The Student's t test was used to assess significance of changes between two groups. A P value < 0.05 was considered statistically significant.

## Results

### STZ induces long-term mechanical allodynia in rats

**Figure 1:**
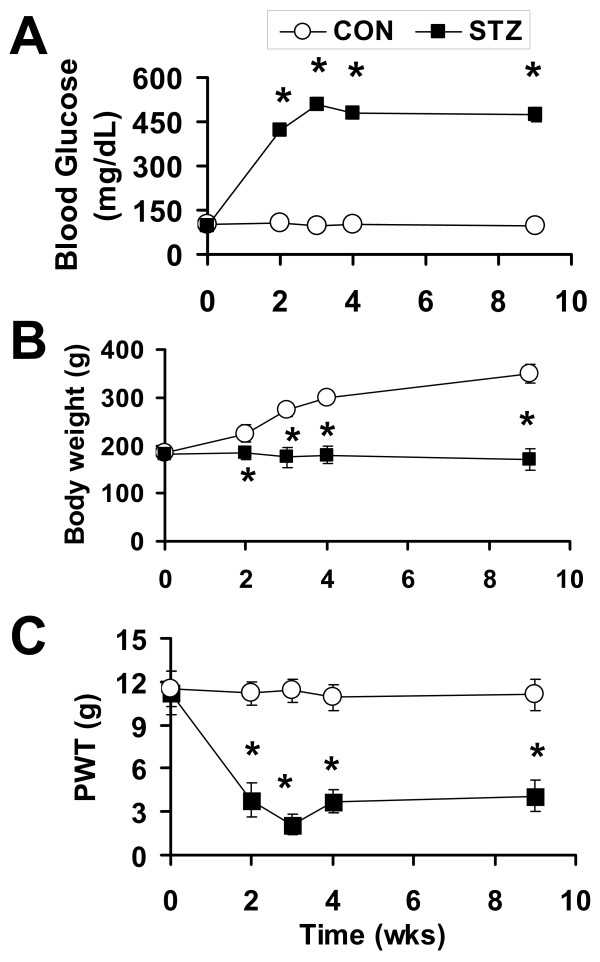
**Hyperglycemia and mechanical allodynia observed in STZ-induced diabetic rats**. (A) Following a single i.p. injection of STZ, the blood glucose level was significantly enhanced two weeks later. The enhancement persisted for at least another 7 weeks. (B) The growth rate of STZ rats was reduced. The body weight of control rats exhibited a steady increase, whereas the body weight of STZ rats remained unchanged. (C) PWTs in response to von Frey filament stimulation were significantly lowered two weeks after the STZ injection. The mechanical allodynia lasted for at least another 7 weeks. Data points are expressed as mean ± S.E.M. Error bars smaller than the size of the symbol are not shown. (*P < 0.05 vs. control, n = 12-15).

STZ has been widely used to induce type 1 diabetes in rodents for the study of pain associated with diabetic neuropathy [4,22,23]. Following a single i.p. injection of STZ (70 mg/kg), we monitored rat blood glucose, body weight and PWT for nine weeks. A majority of the rats (66.7%, n = 68) developed hyperglycemia after STZ treatment. Only rats with a blood glucose concentration > 350 mg/dl were used in further studies. Blood glucose reached the final elevated level two weeks after STZ injection and maintained elevated for at least another 7 weeks (Figure 1A). The diabetic rats also displayed polyuria and an increase in food and water intake (data not shown). Compared with saline-injected rats, the growth rate of STZ rats was noticeably reduced (Figure 1B). In parallel with elevated blood glucose levels, STZ rats also developed mechanical allodynia when tested with von Frey filaments (Figure 1C). The mechanical PWT was 11.2 ± 0.8 g (n = 15) prior to STZ injection, decreased substantially (3.5 ± 0.6 g, n = 15) two weeks after the injection and remained at the reduced level for another 7-week test period (Figure 1C).

### STZ-induced mechanical allodynia is mediated by P2X receptors

**Figure 2:**
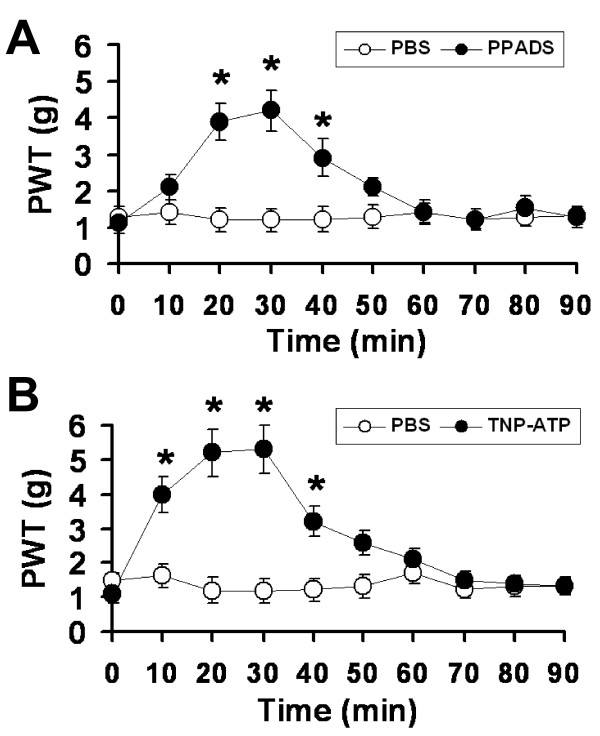
**P2XR antagonists significantly reduce STZ-induced mechanical allodynia**. The effects of the subcutaneously administrated P2XR antagonists (A) PPADS and (B) TNP-ATP on PWTs were studied in rats two weeks after a STZ injection. The PWTs measured in the presence of antagonists were significantly larger than those measured in PBS. Thus, the P2XR antagonists partially reversed the mechanical allodynia induced by the STZ treatment. (*P < 0.05, *n *= 8-10).

To determine if ionotropic P2XRs are involved in the development of allodynia in STZ rats, the P2XR antagonist, PPADS or TNP-ATP was injected into the plantar surface of rat hindpaw and its effects on PWTs were determined. Application of PPADS (25 mmol/50 μl), an antagonist for P2X1, P2X2, P2X3 P2X4 and P2X5 receptors [26], significantly increased PWTs for > 20 min (Figure 2A). In contrast, an application of PBS had no effect on PWTs of STZ-injected rats. To further determine the specific P2XRs in diabetes-induced allodynia, the effect of the potent P2X1, P2X3, P2X2/3 receptor antagonist, TNP-ATP [27], was tested. Following an application of TNP-ATP (50 mmol/50 μl) to the hindpaw of STZ rats, PWTs increased and the effect persisted for > 30 min (n = 8, Figure 2B). Since TNP-ATP has no significant effects on PWTs in control rats [14], those P2XRs do not normally participate in the responses to von Frey filament stimulation in the hindpaw. Taken together, these data suggest that P2X1, P2X3 and/or P2X2/3 receptors are sensitized and involved in the development of diabetes-induced allodynia.

### P2X3R-mediated fast-inactivating ATP currents in hindpaw-innervated DRG neurons are enhanced in STZ-rats

**Figure 3:**
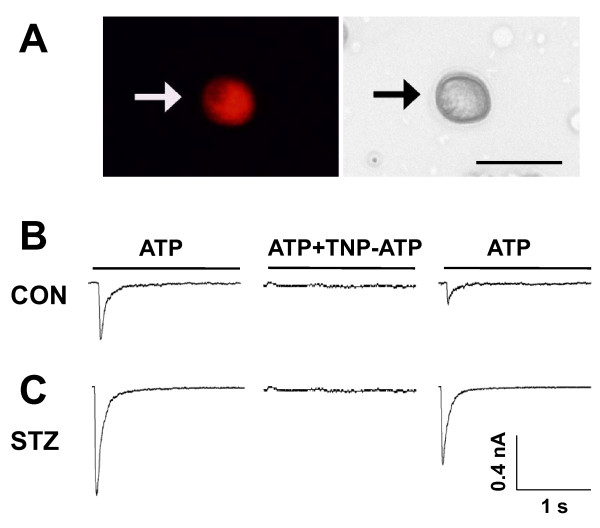
**P2X3R-mediated ATP currents in Dil-labeled DRG neurons**. *A*. An example of a Dil-labeled DRG neuron (Left). A phase image of the same DRG neuron is shown on the right. Examples of fast-inactivating P2X3R-mediated ATP currents evoked in hindpaw-innervated Dil-labeled neurons isolated from L4-6 DRGs of control (*B*) and STZ-treated (C) rats. The *solid line *above each *trace *indicates the period of an application of 20 μM ATP or ATP+TNP-ATP (100 nM). The membrane was held at -60 mV. TNP-ATP completely blocked the fast-inactivating ATP currents obtained from both control and STZ -treated rats. The block was partially reversed following a 3 min washout of TNP-ATP.

**Figure 4:**
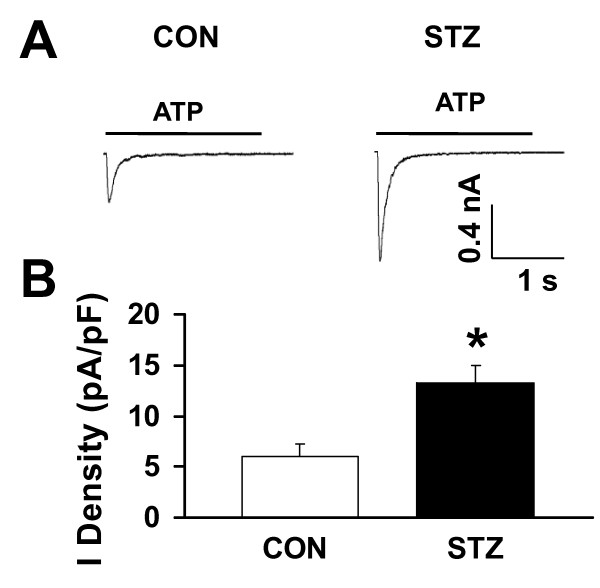
**STZ treatment potentiates fast-inactivating ATP currents**. *A*, Examples of fast-inactivating ATP currents in Dil-labeled DRG neurons isolated from control (*left*) and STZ-treated (*right*) rats. The amplitude of ATP currents obtained from STZ-treated rats was much larger than that obtained in control rats. *B*, Mean peak ATP current densities. The mean peak current density of the fast-inactivating ATP currents measured in DRG neurons of STZ rats was 2.2 times larger than that measured in DRG neurons of control rats (Con, 6.3 ± 1.5 pA/pF, *n *= 15; STZ, 13.8 ± 2.3 pA/pF, *n = *12; *p < 0.05).

We then determined the P2XR-mediated current responses in STZ rats. Since the expression of P2X1R in DRG neurons is low [28], homomeric P2X3 and heteromeric P2X2/3 are likely the P2XRs sensitized by the STZ treatment. ATP-evoked homomeric P2X3R-mediated currents in single lumbar (L4-6) DRG neurons innervating the hindpaw were measured using patch recording techniques. Since lumbar DRGs, e.g., L6 DRG, contain neurons innervating different organs/tissues, including the skin and colon, Dil was injected into the skin of the rat hindpaw immediately prior to the saline or STZ injection. Labeled hindpaw-innervated DRG neurons were identified and recorded 2 weeks later (Figure 3). At holding potential of -60 mV, ATP (20 μM) evoked fast inactivating currents in the majority of DiI-labeled DRG neurons isolated from both control (Figure 3B, left) and STZ-treated rats (Figure 3C, left). The ATP-evoked fast inactivating currents were completely blocked by TNP-ATP (100 nM) in both control and STZ-treated DRG neurons (Figures 3B and 3C, middle), suggesting that the current responses are P2X3R mediated. We then measured the amplitude of P2X3R-mediated ATP currents in DRG neurons and found that the ATP currents were significantly enhanced in DiI-labeled DRG neurons isolated from STZ rats (Figure 4). The average peak current density of P2X3Rs obtained from DRG neurons of STZ rats was 13.6 ± 1.5 pA/pF (*n *= 12), which is 2.3-fold larger than the peak current density, i.e., 6.0 ± 1.3 pA/pF (n = 15), measured in DRG neurons of control rats. Thus, P2X3R-mediated fast-inactivating ATP currents in hindpaw-innervated DRG neurons are greatly potentiated after STZ treatment and contribute to STZ-induced allodynia.

### Membrane expression of P2X3 receptors is upregulated in STZ rats

**Figure 5:**
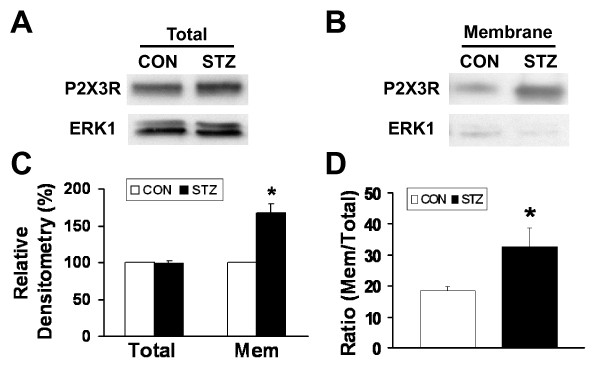
**STZ treatment enhances the membrane protein expression of P2X3Rs**. (A) Total and (B) membrane expression of P2X3R proteins in DRGs of control and STZ-treated rats. The total P2X3R protein expressions were normalized with their respective ERK1. The low expression of ERK1 in membrane fractions suggests a minimal contamination of cytoplasmic P2X3Rs in protein samples. (C) The total expression of P2X3Rs did not change with STZ treatment. On the other hand, the expression of membrane P2X3Rs was significantly increased (*P < 0.05, n = 5). (D) Membrane/total ratio of P2X3R expression. The membrane expression of P2X3Rs was normalized to the total P2X3R expression in its respective rat group. The membrane/total P2X3R expression ratio in control rats was 18.1 ± 0.8% (n = 5) in control rats and was 33.6 ± 5.8% (n = 5) in STZ rats. The membrane expression of P2X3Rs was significantly enhanced with STZ treatment (P < 0.05).

To determine the molecular mechanism underlying the STZ-induced enhancement of ATP currents in DRG neurons, both total and membrane expressions of P2X3Rs in DRGs in control and STZ-treated rat groups were determined using Western blotting analyses two weeks after either a saline or STZ injection. STZ injection did not alter the total P2X3R expression in DRGs (Figures 5A and 5C). On the other hand, the expression of membrane P2X3 receptors was significantly higher in the STZ rat group (Figures 5B and 5C). The membrane/total P2X3R expression ratio increased by 85.6% after STZ treatment (Con: 18.1 ± 0.8%, STZ: 33.6 ± 5.8%, n = 5, *p < 0.05, Figure 5D). Thus, the trafficking of P2X3 receptors to the cell surface membrane in hindpaw-innervated DRG neurons is greatly enhanced in diabetic rats.

## Discussion

In this study, we show that a single injection of STZ can induce diabetes and mechanical allodynia in rats two weeks later. The conditions were maintained for at least another seven weeks (Figure 1). The STZ-induced mechanical allodynia is blocked by the specific P2XR antagonists PPADS and TNP-ATP (Figure 2), an observation in agreement with a previous study of P2XR involvement in a mouse model of diabetes [20]. We further showed that the same dose of ATP evokes much larger P2X3R-mediated inward currents in Dil-labeled hindpaw-innervated DRG neurons isolated from STZ treated rats than those isolated from control rats (Figure 4). The expression P2X3Rs in the cell membrane of STZ primary sensory neurons is significantly upregulated (Figure 5). Thus, P2X3R sensitization is a major contributor to STZ-induced mechanical allodynia. To the best of our knowledge, this is the first report showing an enhancement of P2X3R trafficking to the cell membrane of hindpaw-innervated primary sensory neurons in a painful diabetic rat model.

P2XR sensitization have been implicated in abnormal pain signaling in a variety of injuries [13,29-31], including inflammation [12], sciatic nerve ligation [14,32], burn injury [33] and chronic compression of DRGs [34]. The results of this study and of Migita et al [20] provide the evidence that P2X3R signaling also plays a key role in chronic pain produced by diabetic neuropathy.

TNP-ATP is a potent antagonist for P2X1, P2X3 and P2X2/3 receptors. Because of the low expression of P2X1 receptors in DRGs [13,28,35,36], the complete block of P2X3R-mediated ATP currents by TNP-ATP (Figure 3) and the upregulation of cell membrane trafficking of P2X3Rs in DRG neurons (Figure 5), we conclude that P2X3-containing receptors play a critical role in the development of abnormal pain state following STZ injection. Our study concentrated on diabetes-induced changes in fast-inactivating P2X3R currents. Since P2X2R mRNA was also found to increase after STZ treatment [20], it is of interest to determine, in the future, if slow-inactivating P2X2/3R-mediated currents in Dil-labeled DRG neurons are altered by STZ-induced diabetes. Our conclusion is consistent with the observation that the P2X3R antagonist, A-317491, applied subcutaneously, blocks the mechanical allodynia elicited by nerve injuries [30]. The results also support our previous observation that the P2X3R participates in neuropathic pain in the periphery [14]. In contrast, P2X4/P2X7 receptors in microglia were reported to mediate the neuropathic pain disorders in the spinal cord [37-39].

We showed that TNP-ATP reversed STZ-induced mechanical allodynia partially (Figure 2). The incomplete block of the behavioral responses could be a result of less than saturating concentration of the antagonist used in these experiments. Further experiments are needed to determine the dose response and the maximal effect of the antagonist. In addition, other receptors or channels, e.g. TRPV1 receptors [6,8], T-type Ca^2+ ^channel upregulation could also participate in producing diabetic nociception. In addition to receptor sensitization, an increase in endogenous ATP after STZ treatment could also give rise to the increase in P2XR-mediated allodynia (Figure 2). Our observation that ATP is released from both DRG neurons and glial satellite cells in DRGs [40] have led us to suggest that the origin of ATP release in the hindpaws of STZ rats stems from peripheral nerve terminals and their surrounding glial and immune cells. We measured ATP release in the rat hindpaw following spared nerve injury and found that ATP release was increased only when a stiff (13.5 g) von Frey filament was used for stimulation, but not when a low-strength filament (3.5 g) was applied [14]. Based on the study and the use of low-strength von Frey filaments in diabetic rat experiments (Figure 2), we suggest that an increase in ATP release is not likely the major cause for the observed increase in STZ-induced allodynia. We will test if this is indeed the case in our future study of the role of ATP in diabetes.

The P2X3R expression and trafficking are of great interest in understanding the increase in receptor function under conditions of peripheral inflammation and neuropathy. Changes in the total expression of P2X receptors have been found to vary among nerve injury models. It has been reported that P2X3R expression in DRGs increases after chronic constriction injury [41], decreases when the sciatic or spinal nerves are ligated [42,43], or remains unchanged in spared nerve injury model [14]. The reason for the different observations has not been investigated. The varying findings could simply be a result of different population of neurons in a ganglion affected by different nerve injury models. Migita et al (2009) [20] reported that expression of P2X2 and P2X3 receptor mRNA was upregulated in STZ-induced diabetic mice. They did not study the expression of these receptors at the protein level. Here, we show that STZ treatment does not alter the expression of total P2X3R protein (Figure 5A) but enhances the membrane expression of the receptor (Figure 5C) in rats. In our experiments, the large (2.2-fold) increase in P2X3R-mediated currents following STZ-induced diabetes cannot be entirely accounted for by the increase (85.7%) in P2X3R trafficking. Nevertheless, the relationship between biochemical and electrophysiological or behavioral measurements is not necessarily linear. Other factors, e.g. changes in the modulation of the receptor by protein kinases [24] or changes in channel properties [12], could also contribute to an increase in the P2X3R function. The mechanism underlying the increase in trafficking of P2X3Rs following diabetes has yet to be determined. Our data do indicate that mechanisms involved in P2XR-mediated neuropathic pain and inflammatory pain are distinct. Following CFA-induced peripheral inflammation, the total P2X3R expression has been found to be upregulated [12].

In conclusion, we provide evidence that STZ-induced diabetes promotes the trafficking of P2X3Rs to the membrane of DRG neurons and thus increases P2X3R-mediated responses. The change enhances the activity of afferent neurons and contributes to abnormal diabetes-induced peripheral neuropathic pain. The results may provide promising clues for the development of new therapeutic strategies for managing intractable neuropathic pain in patients with diabetes.

## Competing interests

The authors declare that they have no competing interests.

## Authors' contributions

All authors have read and approved the final manuscript. GYX designed and performed experiments, analyzed data, prepared figures and the manuscript. GL and NAL performed behavioral experiments; LYMH designed experiments and prepared the manuscript.
